# Development and validation of core entrustable professional activities for abdominal radiology

**DOI:** 10.1186/s13244-023-01482-x

**Published:** 2023-08-29

**Authors:** Anita Paisant, Stephen Skehan, Mathilde Colombié, Arthur David, Christophe Aubé

**Affiliations:** 1 Department of Radiology, Abdominal Unit Radiologie A, Angers University Hospital, 4 Rue Larrey, 49933 Angers, France; 2 Laboratoire HIFIH, EA 3859, Université d’Angers, 4 Rue Larrey, 49045 Angers, France; 3 Department of Radiology, Elm Park Dublin 4 and School of Medicine, St Vincent’s University Hospital, University College Dublin, Dublin, Ireland; 4 Department of Radiology, Nantes University Hospital, Hôtel Dieu, 1 Pl. Alexis-Ricordeau, 44093 Nantes Cedex 1, France

**Keywords:** Competency-based education, Curriculum, Education, Digestive system disease, Diagnostic imaging

## Abstract

**Objectives:**

To develop and validate European entrustable professional activities (EPAs) for sub-specialised hepatobiliary and gastrointestinal (HB/GI) diagnostic imaging.

**Materials and methods:**

Both European Society of Radiology and national curricula in HB/GI diagnostic radiology were thoroughly reviewed, resulting in preliminary EPAs drafted by a pilot group of expert radiologists in 2 different countries. Each EPA was fully described with 7 components (Specification/limitations; Potential risks of failing; Relevant domains of competence; Required experience, knowledge, skills, attitude and behaviour; Assessment information sources to assess progress and ground a summative entrustment decision; Entrustment for which level of supervision is to be reached; and Expiration date). The modified Delphi method with 3 Delphi rounds was chosen for validation. Content validity index (CVI) and median values were used for validation.

**Results:**

There were 15 preliminary EPAs, some of them divided according to 2 levels: resident and fellow level. The 37 members of the Delphi group were based in 2 different European countries with a background experience of 10 represented countries. Subsequent to the first Delphi round, 6 EPAs were accepted (CVI ≥ 0.8, median ≥ 4), 6 needed major revisions (CVI 0.7–0.79, median ≥ 4), 3 were rejected (CVI < 0.7) and 1 was added. After the second Delphi round, both the 6 revised EPAs and the additional one met the validation criteria (CVI ≥ 0.8, median ≥ 4). Finally, 13 EPAs were validated during the 3^rd^ Delphi round with an agreement percentage of 95–100%.

**Conclusion:**

This study creates and validates EPAs for sub-specialised HB/GI diagnostic imaging.

**Critical relevance statement:**

Thirteen EPAs for sub-specialised hepatobiliary and gastrointestinal diagnostic imaging were created with a strong methodology, and as a first example set in sub-specialised diagnostic imaging, they provide a template for others to be created.

**Key points:**

• The competence-based teaching in medical studies has recently been reintroduced through EPAs.

• Thirteen EPAs have been developed for hepatobiliary and gastrointestinal sub-specialised diagnostic imaging.

• These EPAs were validated using a Delphi modified method and provide a template for other to be created.

**Graphical abstract:**

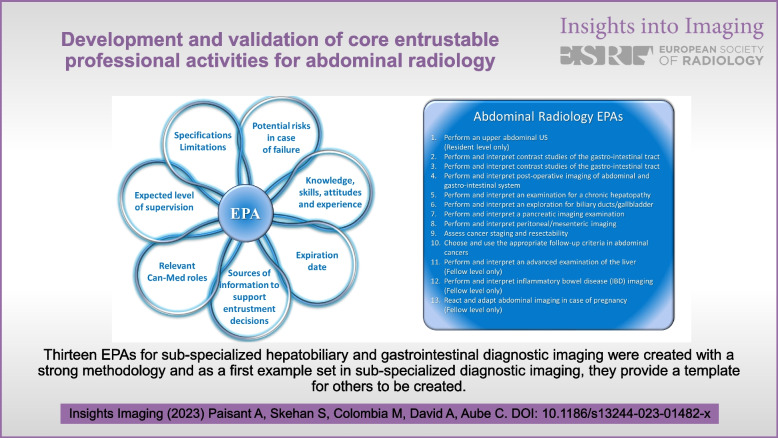

**Supplementary Information:**

The online version contains supplementary material available at 10.1186/s13244-023-01482-x.

## Introduction

Health professional education is mainly based on a learning and knowledge curriculum. The number of publications in the field of radiology has increased exponentially over 40 years. Subsequently, the knowledge curriculum of radiology trainees has widened. However, learning in the workplace must remain part of the trainee’s curriculum. This connection between knowledge and competencies is necessary to optimise medical curricula.

Introducing competency-based education into a trainee curriculum can prove confusing and proper definition is crucial to translate it into daily practice. For this reason, in 2005 Olle ten Cate introduced the concept of entrustable professional activities (EPAs) in medical training to help programme directors and supervisors determine the competence of their trainees [1].

An EPA is a task and/or set of responsibilities that supervisors entrust and delegate to a trainee without supervision, once adequate competence has been obtained [1, 2]. An EPA is a whole unit of professional practice, including several competencies. It must not be confused with a single isolated task (e.g. “perform an MRI with hepatospecific contrast agent”). A full EPA requires 7 components [2–4]:

1. Specification and limitations
2. Potential risks of failing
3. Most relevant domains of competence
4. Required experience, knowledge, skills, attitude and behaviour
5. Assessment information sources to assess progress and ground a summative entrustment decision
6. Entrustment for which level of supervision is to be reached at which stage of training
7. Expiration date

EPAs are an emerging concept and have been recently created in fields such as anaesthesiology and intensive care [5, 6], but rarely, in sub-specialised diagnostic imaging. This is probably due to the difficult task of defining and assessing competencies in pure diagnostic work. In this article, we developed and validated a set of EPAs for hepatobiliary and gastrointestinal diagnostic imaging, using a modified Delphi study based on the method for EPA development previously described by ten Cate and Hennus [6].

## Material and methods

### Development of preliminary EPAs

A pilot group of 4 expert radiologists from two European countries (2 professors and 2 consultants, all specialised in hepatobiliary and gastrointestinal radiology) made an extensive and thorough review of the ESR radiology trainee curriculum. To complement this exhaustive review, the national radiology trainee curricula of two different European countries (France and Ireland) were also reviewed. From these reviews, they identified competencies and created the preliminary set of EPAs. A title and specifications/limitations were defined for each EPA, together with domains of competence, knowledge, skills, attitudes and assessment methods. A preliminary list of 15 EPAs was created, each fully described according the 7 previously described components (see Introduction) [4]. When appropriate, EPA content was divided into resident and fellow level. As explained by ten Cate [4], the conditions for entrustment decisions should guide training activities. Thus, each EPA must also specify expected experience. The modified Delphi method was then used to reach group consensus and to collect expert opinion [7].

### First Delphi round

One month prior to the first Delphi round, all potential participants on the panel were contacted and sent an invitation for the project to be presented. Participants were carefully selected for the Delphi group according to the recommendations for the creation of a Delphi panel [7–11]. A wide and representative range of participants from two European countries was contacted to be part of the Delphi group, including professors, consultants, fellows and residents. A preparatory video-conference session, explanatory email and reference articles for EPA were sent to participants. This one-month delay was chosen so that all participants had enough time to contact the pilot group for more explanations if needed.

An online survey was developed for the first Delphi round [12]. An example of the survey can be seen in Additional file 1. The survey was tested by three radiologists from the two countries (one professor, one consultant and one resident) in order to ensure i. clarity and format of the questions and ii. comprehensiveness of the questions such that an appropriate answer could be given. These three radiologists did not take part to the Delphi tour. Some minor textual revisions were made following the survey testing. The electronic survey was then sent to 48 stakeholders, along with a second detailed email containing again (repetitive) information on EPAs.

Panel members were asked to score each EPA for “indispensability”, “comprehensiveness/clarity” and “completeness” with a 5-point Likert-scale as follows: 5, Strongly agree—4, Agree—3, Neither agree or disagree—2, Disagree—1, Strongly disagree. This 5-point Likert-scale was chosen with the intention that the mid-point represents a neutral response. In order not to misinterpret the words, digits from 1 to 5 were also added [13]. After each answer, an open text box was available for any additional suggestion, including an additional suggested EPA. In addition, all preliminary listed knowledge, skills and attitudes were given point-by-point approval with a 2-point binary scale: 2, approve—1, disapprove. Again, open text boxes were provided following each section for free text additional suggestions. The final part of the survey asked for the number of successfully completed examinations for entrustment (open box for numbers), expected level of supervision (scale from 1 to 5) for both resident and fellow level, and the expiration date (open box for numbers). The levels of supervision are:

- Level 1: Not allowed to practice EPA
- Level 2: Allowed to practice EPA only under proactive, full supervision
- Level 3: Allowed to practice EPA only under reactive/on-demand supervision
- Level 4: Allowed to practice EPA unsupervised
- Level 5: Allowed to supervise others in practice of EPA

### Data analysis of the first Delphi round

After the first Delphi round, all results and comments were analysed by the 4 members of the pilot group. First, content validity index (CVI) of each Likert-scale was calculated for each EPA [14]. For each item, the CVI is computed as the number of experts giving a rating of either 4 or 5, divided by the number of experts. CVI was originally described with a 4-point rating scale [14, 15]. As a five-point Likert-scale was chosen for our study, the management of CVI results was as previously described in the study of Hennus et al. [6]: a CVI of 0.8 or higher indicated sufficient content validity, a CVI within the range 0.70 and 0.79 implied that the item required revision, and a CVI below 0.70 indicated elimination of the corresponding EPA. The median score of each item (indispensability, comprehensiveness/clarity and completeness) was then calculated, and a median < 4 was deemed to indicate that the EPA as needed revisions. For questions involving a 2-point scale, the item was approved if CVI of 0.8 or higher and deleted if under 0.8. Comments and suggestions in open text box were all reviewed by the 4 members of the pilot group and dealt with as follows: (a) suggestion regarding textual clarifications and/or alterations: suggestion accepted if unanimously agreed on by the pilot group; (b) suggestion contradicting existing EPA guidelines: suggestion rejected; (c) suggestion regarding content of an EPA made by > 5% of all panellists: suggestion accepted; and (d) suggestion regarding content of an EPA made by < 5% of all panellists: suggestion rejected. For the number of successfully completed examinations for entrustment, the median value was considered. For expected level of supervision and expiration date, the mean value was considered. Data were analysed using SPSS software, version 15.0.

### Second Delphi round

All results from the first Delphi round were summarised and sent to the Delphi panellists. Emphasis was placed on clear and easy visualisation of the results and modifications, using comparison tables, graphs and colours. Retained additional suggestions were presented with the percentage of the panellists suggesting them. The survey of the second Delphi round included only EPAs needing revisions according to CVI and median value. The panellists were again asked to score each revised EPA for “indispensability”, “comprehensiveness/clarity” and “completeness” with a 5-point Likert-scale. Knowledge, skills and attitudes were rated for global approval with a 2-point binary scale. In addition, one EPA was added to the survey (EPA 16, see Result section). This EPA underwent the same process as the first Delphi round.

### Data analysis of the second Delphi round

CVI and median values were calculated for each scale. All new results and comments were again analysed by the 4 members of the pilot group. The prior data analysis described in the first Delphi round was conducted, both with the revised EPA and the new one. Data were analysed using SPSS software, version 15.0.

### Third Delphi round

For this last round, each EPA was presented as a card with the entire 7 components completed as a final version. The panellist was asked for global approval with a 2-point binary scale “agree” or “disagree” for each EPA, and for approval for implementation of the whole EPA set into the medical imaging curriculum.

## Results

### First Delphi round

**Fig. 1:**
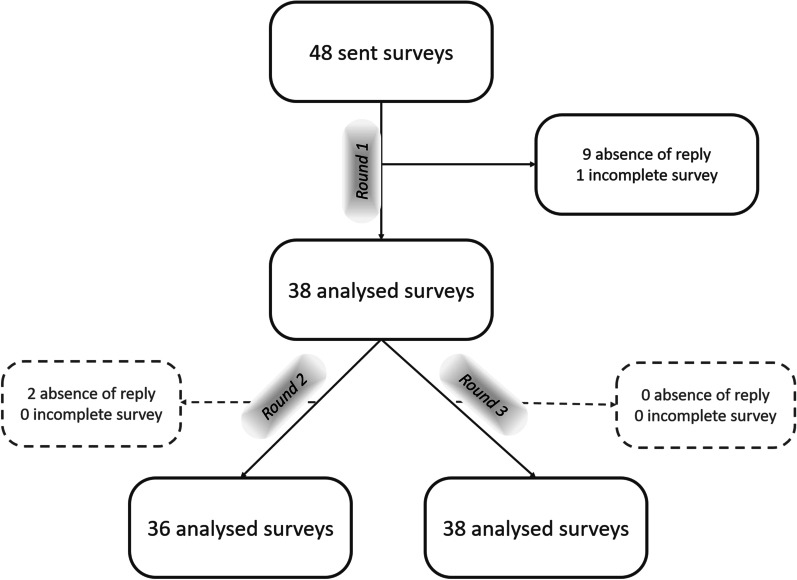
Flow chart of the study

From the 48 surveys sent to panellists, 38 complete responses (79%) from two different countries were received (Fig. 1). The final panel included 15 professors (39.5%), 15 consultants (39.5%, 13 public and 2 private), 5 fellows (13%) and 3 residents (8%). All participants were sub-specialised in hepatobiliary and gastrointestinal imaging. (The 3 residents had experience in hepatobiliary and gastrointestinal imaging.) Twenty-three (60%) of the participants were male and fifteen (40%) female (Table 1). From the 38 participants, 13 had prior experience of sub-specialised hepatobiliary and gastrointestinal imaging in other countries for at least 6 months (mean = 2 years ± 2.7). In total, 10 different countries were represented.

**Table 1:** Panellist characteristics of the first Delphi round

	N = 38
*Gender (%)*	
Male	23 (60)
Female	15 (40)
Age (mean ± SD)	42 ± 11
*HB/GI activity*	
Diagnostic only	14
Diagnostic and interventional	24
*Years of experience*	
< 5	13
5–19	14
≥ 20	11
*Grade*	
Professor (%)	15 (38)
Consultant (%)	15 (40)
Fellow (%)	5 (14)
Resident (%)	3 (8)

**Fig. 2:**
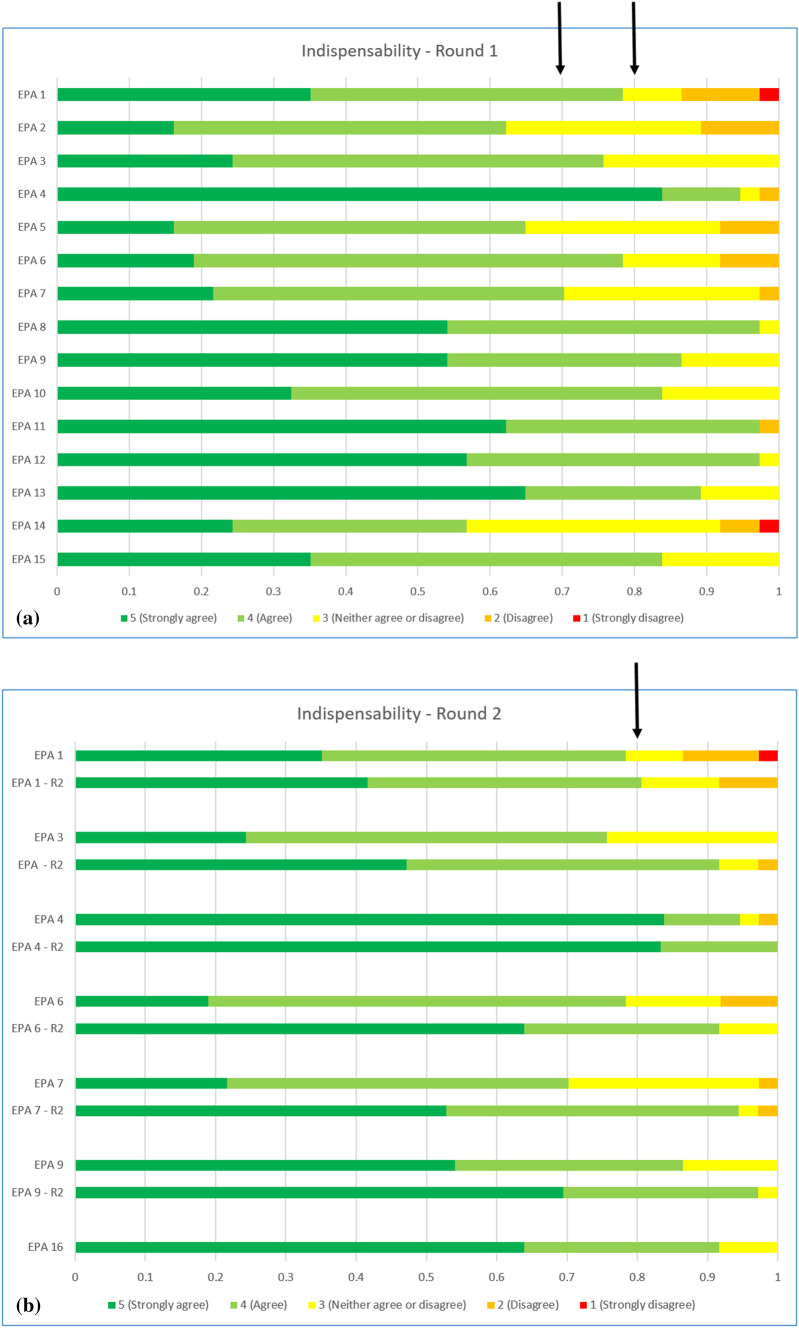
Results for indispensability of the first (**a**) Delphi round and comparison with the second (**b**) Delphi round. EPA 2, 5 and 14 are eliminated from the second round as content validity index (CVI) for indispensability was below 0.7. EPA 16 was added in the second Delphi round

**Fig. 3:**
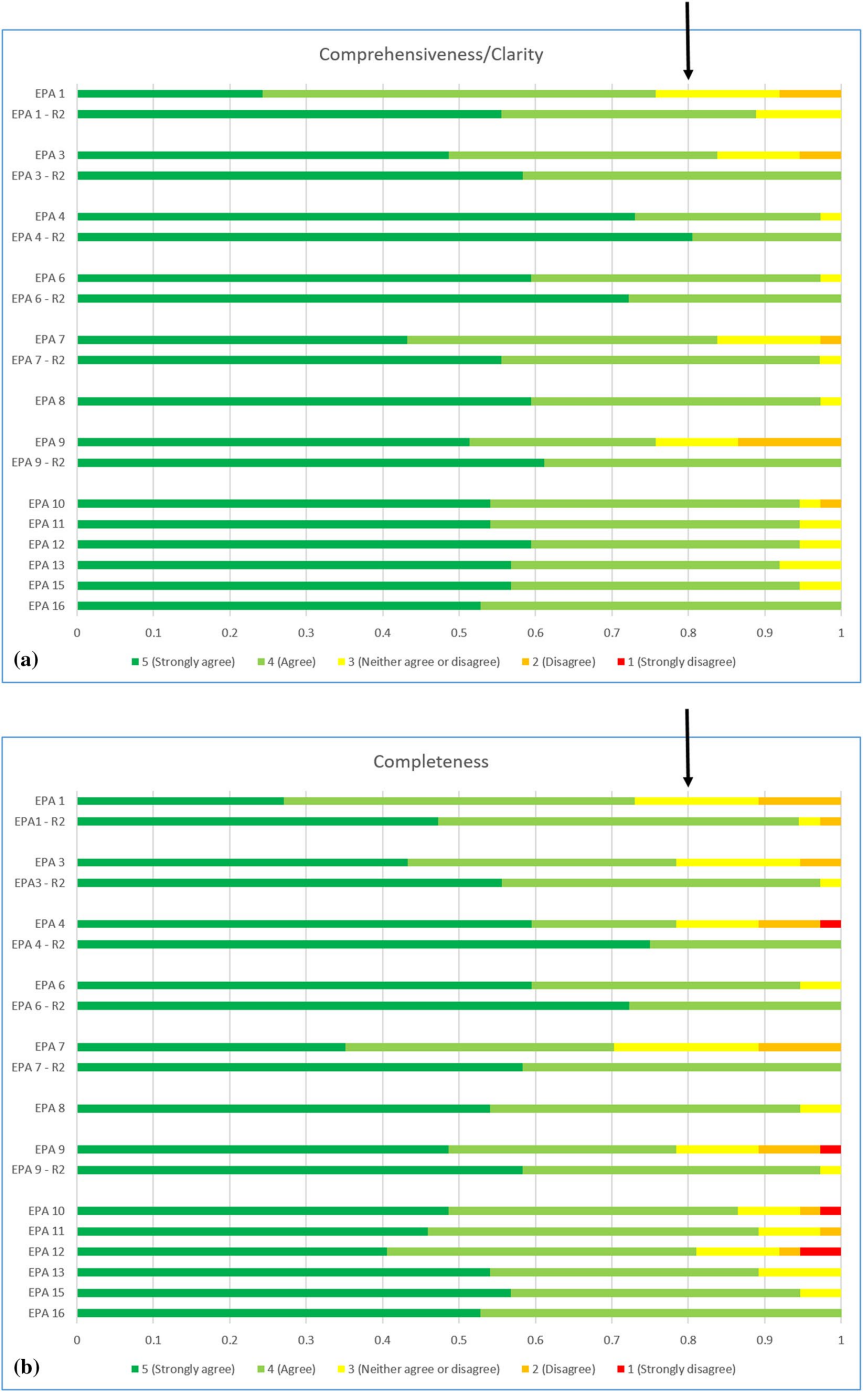
Results of comprehensiveness/clarity (**a**) and completeness (**b**) of the first Delphi round and comparison with the revised EPA of the second Delphi round. EPA 2, 5 and 14 have been previously eliminated due to content validity index (CVI) for indispensability below 0.7 and are not represented

The content validity index (CVI) and median value were calculated for indispensability, comprehensiveness/clarity and completeness (Table 2, Figs. 1, 2 and 3). All median values were ≥ 4. Six EPAs had CVI > 0.8 and were accepted (Fig. 2a). Six EPAs (EPA 1, EPA 3, EPA 4, EPA 6, EPA 7 and EPA 8) did not meet the threshold of 0.8 for either “indispensability”, “comprehensiveness/clarity” and/or “completeness”, with CVI ranging from 0.7 to 0.8. These EPAs underwent major revisions according to the comments associated to each results. Three EPAs had CVI < 0.7 for indispensability and were rejected. The three rejected EPAs were: “Perform and interpret a specific colonic examination”, “Perform and interpret oesophageal imaging” and “Effectively contribute clinical/imaging opinion to abdominal/digestive multidisciplinary team (MDT) meetings” (see Discussion section).

**Table 2:** Content validity index (CVI) of indispensability, comprehensiveness/clarity and completeness for each EPA according to Delphi round 1 and 2

	CVI indispensability		CVI comprehensiveness/clarity		CVI completeness		Agreement (%)
	Round 1	Round 2	Round 1	Round 2	Round 1	Round 2	Round 3
1	**0.78**		**0.76**	0.89	**0.73**	0.94	100
2	*0.62*						
3	**0.76**	0.92	0.84	1.00	**0.78**	0.97	97
4	0.95	1.00	0.97	1.00	**0.78**	1.00	100
5	*0.65*						
6	**0.78**	0.92	0.97	1.00	0.95	1.00	100
7	**0.70**	0.94	0.84	0.97	**0.70**	1.00	97
8	0.97		0.97		0.95		100
9	0.86	0.97	**0.76**	1.00	**0.78**	0.97	95
10	0.84		0.95		0.86		97
11	0.97		0.95		0.89		100
12	0.97		0.95		0.81		95
13	0.89		0.92		0.89		97
14	*0.57*						
15	0.84		0.95		0.97		97
16		0.97		1.00		0.97	100

Final agreement percentage for Delphi round 3

Bold: require revisions

Italics: elimination of the corresponding EPA

Moreover, 11% of the panellist asked for an additional EPA (“Perform and interpret post-operative imaging of abdominal and gastrointestinal system”) that was added in round 2 (EPA 16).

### Second Delphi round

From the 38 sent surveys, there were 36 (95%) responses, all fully completed. The revised EPAs all improved their CVI > 0.8 for indispensability, comprehensiveness/clarity and completeness (Figs. 2b, 3, Table 2). All median values were ≥ 4. The additional EPA 16 was directly approved for round 3 as CVI was over 0.8 for indispensability, comprehensiveness/clarity and completeness. All knowledge, skills and attitudes of this EPA were also approved with CVI > 0.8.

### Third Delphi round

**Fig. 4:**
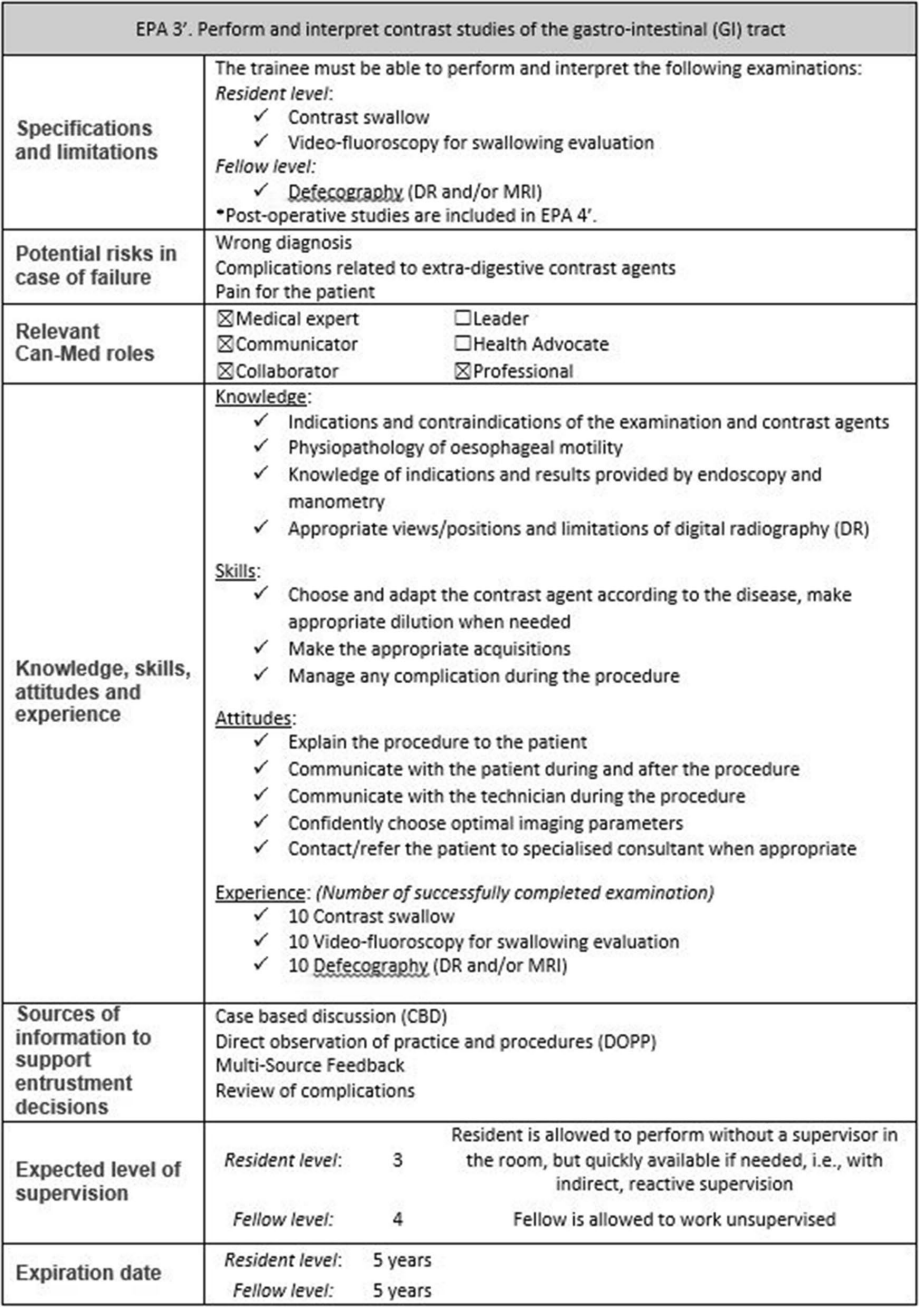
Example of EPA 3’ for the trainee curriculum with the 7 components

From the 38 sent surveys, there were 38 (100%) complete responses. All EPAs were validated by the panel (Table 2). The agreement for implementation of the whole EPA set into the medical imaging curriculum was 92%. Subsequent to the comments of the panellists, the order of the EPAs was changed in the final presentation, so that EPAs covering more basic resident-level competencies were placed at the beginning of the curriculum and more advanced fellow-level EPAs were placed towards the end of the curriculum. The table of correspondence can be seen in Supplementary materials 2. There are 13 final EPAs for hepatobiliary and gastrointestinal diagnostic imaging, all summarised in Table 3. Figure 4 shows an example of one complete EPA. All complete EPAs are accessible in Supplementary materials 3.

**Table 3:** The final EPAs for hepatobiliary and gastrointestinal diagnostic imaging trainees

EPA 1’	Perform an upper abdominal US (Resident level only)
EPA 2’	Perform and interpret an abdominal wall examination (US and CT) (Resident level only)
EPA 3’	Perform and interpret contrast studies of the gastrointestinal (GI) tract
EPA 4’	Perform and interpret post-operative imaging of abdominal and gastrointestinal system
EPA 5’	Perform and interpret an examination for a chronic hepatopathy
EPA 6’	Perform and interpret an exploration for biliary ducts/gallbladder
EPA 7’	Perform and interpret a pancreatic imaging examination
EPA 8’	Perform and interpret peritoneal/mesenteric imaging
EPA 9’	Assess cancer staging and resectability
EPA 10’	Choose and use the appropriate follow-up criteria in abdominal cancers
EPA 11’	Perform and interpret an advanced examination of the liver (Fellow level only)
EPA 12’	Perform and interpret inflammatory bowel disease (IBD) imaging (Fellow level only)
EPA 13’	React and adapt abdominal imaging in case of pregnancy (Fellow level only)

## Discussion

A competency-based curriculum is lacking in sub-specialised diagnostic imaging, probably due to the difficult task of defining competency in a purely diagnostic, intellectual activity, compared, for example, with a procedure-based activity. In our study, we developed a list of 13 EPAs for hepatobiliary and gastrointestinal diagnostic imaging. The study was validated with an international panel, using a strong methodology: the modified Delphi method.

Apart from the method, one of the main strengths of our study is the wide validation of the content of each EPA. Not only were panellists asked to assess indispensability, comprehensiveness/clarity and completeness, but also to validate the knowledge, skill and attitude necessary for each EPA, as well as the required experience for each examination, level of supervision according to the stage of training (resident versus fellow) and expiration date. This level of validation and its method have not previously been reported, to our knowledge, in the literature.

Our study went further than the previous studies on EPA, with the subdivision of hepatobiliary and gastrointestinal diagnostic imaging curriculum into resident and fellow levels. This approach was difficult to consider for the pilot group and was potentially confusing for the panellist. Careful and precise explanation was given to each panellist prior to the study for everyone to have the same definition. A few additional questions were answered during the first Delphi round when asked by individual stakeholders. Moreover, many comments in the open text boxes referred to the attributed level of competencies. The resident/fellow levels were initially created according to the European/national curricula. So, when explicitly specified in these curricula, they were not changed and this was explained to panellists who had suggested a change, before the second round. However, for some competencies, the level of trainee was not explicitly specified. In that case, the comments were taken into consideration following the rules described in Materials and methods.

The three rejected EPAs after the first Delphi round were: i. perform and interpret a specific colonic examination; ii. perform and interpret oesophageal imaging; and iii. effectively contribute clinical/imaging opinion to abdominal/digestive multidisciplinary team (MDT) meetings. With CVI = 0.62 and CVI = 0.65 for indispensability, respectively, the two first EPAs were rejected by the panel based on similar arguments such as requiring much too specific skills, along with low throughput of examinations and consequent difficulty teaching them in many university centres, and thus should not be part of the systematic curriculum of each trainee (16% and 19% of the panellist, respectively). However, this rejection as part of systematic curriculum of the trainee should not discourage centres from trying to teach these examinations when possible for them and when the trainee is interested in learning. The third rejected EPA concerning competencies at MDT had the lowest CVI of 0.57 with the largest amount of comments against that EPA, arguing that this EPA was neither resident nor fellow level. The pilot group was first surprised by this decision of the panel, as participation at MDTs was part of the 3 curricula they reviewed. However, going back to the definition of an EPA, the panel was actually correct in its assessment, because an EPA is defined as a task that supervisors delegate to a trainee to perform unsupervised once adequate competence has been obtained [4]. However, in the ESR curriculum level II, for example, this competence/attitude was defined as “To participate in and to perform under supervision at multidisciplinary conferences”. Indeed, 22% of the panellists clarified their rejection by stating that trainees must participate in MDTs, but delegation should not be considered before becoming a consultant. This example also shows the strong methodology of the Delphi method.

This study has several limitations, mainly related to the Delphi group participants. The Delphi survey is a group designed to transform opinion into group consensus. The members of the Delphi group should be individuals who have knowledge of the topic under investigation, defined as “panel of informed individuals” or “experts” [9, 10]. Our panel members were selected for their knowledge of and commitment to abdominal radiology. The majority of them worked in university medical centres. This may have biased some of the results, but we tried to balance the panel by choosing panellists from a variety of backgrounds and levels of expertise, including professors, consultants, fellows and residents. In addition, although we tried to develop these EPAs for inclusion in the European trainee curriculum, the members of the Delphi group were only from two specific countries. Although the European Society of Radiology curriculum tends to provide homogeneous recommendations across European countries, many countries also have their own national curriculum, which may differ slightly from country to country. Therefore, the implementation of these EPAs in different European environments is potentially challenging. Further studies could validate the results in a larger number of countries. It is also very likely that each country will have its own guidelines for some specific topics, depending on the structure of the health system and local practices. The inclusion of additional guidance and information on these recommendations (e.g. the purpose of the EPAs) would likely also help their implementation by building confidence in the ability to use EPAs [16, 17].

In conclusion, this study developed and validated a set of 13 EPAs which could be used as a European trainee curriculum for hepatobiliary and gastrointestinal diagnostic imaging. The robust methodology and European validation make it widely applicable and offer a potential template for EPA creation in other sub-specialities in diagnostic imaging.

### Supplementary Information

**Additional file 1: **ESM 1: Example of survey for EPA 3. First Delphi round. ESM 2: Table of correspondence between EPA figures of the 3 Delphi rounds and the final presented EPA. ESM 3: The 13 EPAs for sub-specialised HB/GI diagnostic imaging.

## Data Availability

The datasets used and/or analysed during the current study are available from the corresponding author on reasonable request.

## Abbreviations

CVI: Content validity index
EPAs: Entrustable professional activities
