# Supplementary material for: Development and validation of core entrustable professional activities for abdominal radiology
Source: Insights Imaging. 2023 Aug 29;14:142. doi: 10.1186/s13244-023-01482-x (PMC10465426; doi:10.1186/s13244-023-01482-x)
Supplement: Supplementary file 1 — Additional file 1: ESM 1: Example of survey for EPA 3. First Delphi round. ESM 2: Table of correspondence between EPA figures of the 3 Delphi rounds and the final presented EPA. ESM 3: The 13 EPAs for sub-specialised HB/GI diagnostic imaging. [file 13244_2023_1482_MOESM1_ESM.pdf]

Development and validation of core entrustable professional activities for abdominal radiology

ELECTRONIC SUPPLEMENTARY MATERIAL

ESM 1

Example of survey for EPA 3. First Delphi round.

EPA3. ***Perform and interpret an advanced examination of the liver***

The trainee must be able to perform and interpret the following examinations:

- *Hepatic graft examination (US/CT)*
- *Rendu-Osler/ Budd-Chiari examination (US/CT)*
- *CEUS of a liver lesion*
- *US/CEUS of the liver with fusion*
- *Elastography of the liver (US/MRI)*
- *Liver quantification in MRI (iron, steatosis)*
- *Hepatic volumetry*
- *MRI with hepatospecific contrast agent (Primovist and Multihance (Multihance France only))*

For EPA 3, please indicate whether you regard it as indispensable for the student curriculum.

Strongly disagree - Disagree - Neither agree nor disagree - Agree - Strongly agree

	Strongly disagree 1	2	3	4	Strongly agree 5
EPA 3: Perform and interpret an advanced examination of the liver.	○	○	○	○	○

Reset

If you have answered strongly disagree/disagree/neither agree nor disagree to any of the EPAs, please provide a short explanation below.

Characters Remaining: 500

For EPA 3, indicate the comprehensiveness/clarity.

Strongly disagree - Disagree - Neither agree nor disagree - Agree - Strongly agree

	Strongly disagree				Strongly agree
	1	2	3	4	5
**EPA 3: Perform and interpret an advanced examination of the liver.**	○	○	○	○	○

Reset

If you have answered strongly disagree/disagree/neither agree nor disagree, please provide additional proposition below.

For EPA 3, indicate if it's complete.

Strongly disagree - Disagree - Neither agree nor disagree - Agree - Strongly agree

	Strongly disagree				Strongly agree
	1	2	3	4	5
**EPA 3: Perform and interpret an advanced examination of the liver.**	○	○	○	○	○

Reset

If you have answered strongly disagree/disagree/neither agree nor disagree, please provide additional proposition below.

For each knowledge, please indicate whether it is appropriate for this EPA.

	Disapprove<br>1	Approve<br>2
Physics basis of elastography and iron/steatosis overload	○	○
Indications for CEUS/elastography/fat/iron quantification	○	○
Performance and limits of elastography, iron/fat quantification	○	○
Surgical techniques of hepatic graft	○	○
Normal and abnormal US values in hepatic graft	○	○
Rare hepatic vascular disease physiopathology and complications	○	○
Pathophysiology of Primovist and Multihance (Multihance France only)	○	○

Reset

Are there additional knowledges that you think should be included? If yes, describe them. Please also provide any additionnal proposition if needed.

For each skill, please indicate whether it is appropriate for this EPA.

	Disapprove<br>1	Approve<br>2
Adapt CEUS injection to the suspected hepatic lesion	○	○
Set up and improve MRI sequences to make a diagnosis	○	○
Use appropriate software (quantification, volumetry)	○	○
Be competent in performing US liver with fusion	○	○

Reset

Are there additional skills that you think should be included? If yes, describe them. Please also provide any additionnal proposition if needed.

For each attitude, please indicate whether it is appropriate for this EPA.

	Disapprove<br>1	Approve<br>2
Look for the patient's history when necessary (graft surgery)	○	○
Adapt examination for a proper diagnosis if there are technical limitations	○	○
Search in the literature for new studies and guidelines	○	○
Communicate comprehensive results in the report	○	○

Reset

Are there additional attitudes that you think should be included? If yes, describe them. Please also provide any additionnal proposition if needed.

What is the number of successfully completed examination that should be done?

Hepatic graft examinations US

Hepatic graft examinations CT

Rendu-Osler examinations US

Rendu-Osler examinations CT/MRI

Budd-Chiari examinations US

Budd-Chiari examinations MRI

CEUS of a liver lesion

US/CEUS of the liver with fusion

Elastography of the liver US

Elastography of the liver MRI

Liver quantification in MRI (iron, steatosis)

Hepatic volumetry

MRI with hepatospecific contrast agent (Primovist and Multihance)

What do you think should be the expected level of supervision?

- 3 (the learner is allowed to carry out the EPA without a supervisor in the room, but quickly available if needed, i.e., with indirect, reactive, supervision)
- 4 (the learner is allowed to work unsupervised)
- 5 (the learner is allowed to provide supervision to more junior learners)

What do you think should be the expiration period (in years)?

ESM 2

Table of correspondence between EPA figures of the 3 Delphi rounds and the final presented EPA.

Final EPA figure	Original EPA figure for the study
EPA 1’	EPA 4
EPA 2’	EPA 6
EPA 3’	EPA 1
EPA 4’	EPA 16
EPA 5’	EPA 8
EPA 6’	EPA 11
EPA 7’	EPA 12
EPA 8’	EPA 7
EPA 9’	EPA 9
EPA 10’	EPA 15
EPA 11’	EPA 3
EPA 12’	EPA 10
EPA 13’	EPA 13

ESM 3

EPA 1'. Perform and interpret an upper abdominal US (Resident level only)			
**Specifications and limitations**	The trainee must be able to perform a US of the following structures:<br>✓ The liver (Doppler evaluation of the hepatic vessels is included.)<br>✓ The gallbladder<br>✓ The bile ducts (intra and extra hepatic)<br>✓ The pancreas<br>✓ The spleen<br>✓ The kidneys<br>✓ The aorta (includes morphology, not Doppler)		
**Potential risks in case of failure**	Wrong diagnosis<br>Delay in diagnosis<br>Pain, infection, tumour/thrombus extension		
**Relevant Can-Med roles**	☒Medical expert ☐Leader<br>☒Communicator ☐Health Advocate<br>☒Collaborator ☒Professional		
**Knowledge, skills, attitudes and experience**	<u>Knowledge</u>:<br>✓ Anatomy and physiology of the liver, gallbladder, bile ducts, pancreas, kidneys and spleen<br>✓ Limitations of US for each organ and alternative examinations<br>✓ Normal values and appearance<br>✓ Physics basis of Doppler US<br>✓ Normal and abnormal US of hepatic vessels<br><u>Skills</u>:<br>✓ Perform a normal/abnormal US of the upper abdomen<br>✓ Confidently choose optimal imaging parameters for B-mode and colour Doppler<br>✓ Perform a portal, arterial and hepatic vein examination with Doppler US<br><u>Attitudes</u>:<br>✓ Communicate with the patient during and after the examination<br>✓ Present the results to the patient<br>✓ Propose further action in case of pathology or technical limitation<br>✓ Confidently choose optimal imaging parameters<br><br><u>Experience</u>: *(Number of successfully completed examination)*<br>✓ 50 US		
**Sources of information to support entrustment decisions**	Direct observation of practice and procedures (DOPP)<br>Multi-Source Feedback		
**Expected level of supervision**	*Resident level:*	4	Resident is allowed to work unsupervised
**Expiration date**	*Resident level:*	4 years	

EPA 2’. Perform and interpret an abdominal wall examination (US and CT) (Resident level only)			
**Specifications and limitations**	The resident must be able to perform and interpret an imaging examination to explore abdominal wall pathology and complications (Resident level). Imaging modalities include US and CT.		
**Potential risks in case of failure**	Wrong diagnosis<br>Complications related to extra-digestive contrast agents<br>Pain for the patient		
**Relevant Can-Med roles**	☒Medical expert ☐Leader<br>☒Communicator ☐Health Advocate<br>☒Collaborator ☒Professional		
**Knowledges, skills, attitudes and experience**	Knowledge:<br>✓ Different types of hernia of the abdominal wall<br>✓ Potential complications of abdominal wall hernia<br>✓ Diagnosis limitations for each technique<br>Skills:<br>✓ Adapt the patient’s position when needed according to the clinical query (particularly at US examination)<br>✓ Ask the patient to perform a Valsalva manoeuvre when needed<br>✓ Use contrast injection during CT only when necessary<br>✓ Choose the most appropriate imaging examination and adapt it according to the clinical problem<br>Attitudes:<br>✓ Communicate and explain the result to the patient (US)<br>Experience: *(Number of successfully completed examination)*<br>✓ 15 US<br>✓ 10 CT		
**Sources of information to support entrustment decisions**	Case based discussion (CBD)<br>Direct observation of practice and procedures (DOPP)<br>Multi-Source Feedback<br>Clinical Work Sampling (CWS) for In-Training Evaluation<br>Case Presentation (CP)		
**Expected level of supervision**	*Resident level:*	3	Resident is allowed to perform without a supervisor in the room, but quickly available if needed, i.e., with indirect, reactive, supervision
**Expiration date**	*Resident level:*	5 years	

EPA 3'. Perform and interpret contrast studies of the gastro-intestinal (GI) tract	
**Specifications and limitations**	The trainee must be able to perform and interpret the following examinations:<br>*Resident level*:<br>✓ Contrast swallow<br>✓ Video-fluoroscopy for swallowing evaluation<br>*Fellow level:*<br>✓ Defecography (DR and/or MRI)<br>*Post-operative studies are included in EPA 4'.
**Potential risks in case of failure**	Wrong diagnosis<br>Complications related to extra-digestive contrast agents<br>Pain for the patient
**Relevant Can-Med roles**	☒Medical expert ☐Leader<br>☒Communicator ☐Health Advocate<br>☒Collaborator ☒Professional
**Knowledge, skills, attitudes and experience**	<u>Knowledge</u>:<br>✓ Indications and contraindications of the examination and contrast agents<br>✓ Physiopathology of oesophageal motility<br>✓ Knowledge of indications and results provided by endoscopy and manometry<br>✓ Appropriate views/positions and limitations of digital radiography (DR)<br><br><u>Skills</u>:<br>✓ Choose and adapt the contrast agent according to the disease, make appropriate dilution when needed<br>✓ Make the appropriate acquisitions<br>✓ Manage any complication during the procedure<br><br><u>Attitudes</u>:<br>✓ Explain the procedure to the patient<br>✓ Communicate with the patient during and after the procedure<br>✓ Communicate with the technician during the procedure<br>✓ Confidently choose optimal imaging parameters<br>✓ Contact/refer the patient to specialised consultant when appropriate<br><br><u>Experience</u>: *(Number of successfully completed examination)*<br>✓ 10 Contrast swallow<br>✓ 10 Video-fluoroscopy for swallowing evaluation<br>✓ 10 Defecography (DR and/or MRI)
**Sources of information to support entrustment decisions**	Case based discussion (CBD)<br>Direct observation of practice and procedures (DOPP)<br>Multi-Source Feedback<br>Review of complications
**Expected level of supervision**	*Resident level*: 3 — Resident is allowed to perform without a supervisor in the room, but quickly available if needed, i.e., with indirect, reactive supervision<br>*Fellow level:* 4 — Fellow is allowed to work unsupervised

**Expiration date**	*Resident level*:	5 years
	Fellow level:	5 years

<table>
<tr><th colspan="2">EPA 4’. Perform and interpret post-operative imaging of abdominal and gastro-intestinal system</th></tr>
<tr><td>Specifications and limitations</td><td>The trainee must be able to perform and interpret the following examinations:
Resident level:
✓ Post-surgery studies of the abdominal wall
✓ Post-surgery studies of the oesophagus and colon/rectum. This includes opacification (CT and/or digital radiography (DR))
Fellow level:
✓ Post-surgery studies following hepato-biliary surgery
✓ Post-surgery studies following pancreatic surgery
✓ Post-surgery studies following bariatric surgery</td></tr>
<tr><td>Potential risks in case of failure</td><td>Wrong diagnosis and subsequent complications
Complications related to extra-digestive contrast agents
Pain for the patient</td></tr>
<tr><td>Relevant Can-Med roles</td><td>☒Medical expert ☐Leader
☒Communicator ☐Health Advocate
☒Collaborator ☒Professional</td></tr>
<tr><td>Knowledge, skills, attitudes and experience</td><td><u>Knowledge</u>:
✓ Indications and contraindications of the examination and contrast agent
✓ Surgical technique and normal post-op appearances
✓ Complicated post-op appearances
✓ Appropriate views/positions and limitations of digital radiography (DR)
✓ Limits of each technique
<u>Skills</u>:
✓ Choose the most appropriate imaging examination and adapt it according to the clinical problem
✓ Choose and adapt the contrast agent according to the disease, make appropriate dilution when needed
✓ Catheterise a stoma for opacification and perform pouchograms and loopograms
✓ Make the appropriate acquisitions
✓ Manage any complication during the procedure
<u>Attitudes</u>:
✓ Explain the procedure and communicate with the patient before, during and after the procedure
✓ Supervise and teach technical staff to ensure that appropriate images are obtained
<u>Experience</u>: (Number of successfully completed examination)
✓ 10 Post-surgery studies of the abdominal wall
✓ 20 Post-surgery studies of the GI system, including colon/rectum opacification (CT and/or digital radiography (DR))
✓ 20 Post-surgery studies following hepato-biliary surgery
✓ 15 Post-surgery studies following pancreatic surgery
✓ 20 Post-surgery studies following bariatric surgery</td></tr>
<tr><td>Sources of information to support entrustment decisions</td><td>Case based discussion (CBD)
Direct observation of practice and procedures (DOPP)
Case Presentation (CP)</td></tr>
</table>

**Expected level of supervision**	*Resident level*: 3 — Resident is allowed to perform without a supervisor in the room, but quickly available if needed, i.e., with indirect, reactive, supervision<br>*Fellow level:* 4 — Fellow is allowed to work unsupervised
**Expiration date**	*Resident level*: 4 years<br>*Fellow level:* 4 years
EPA 5'. Perform and interpret an examination for a chronic hepatopathy	
**Specifications and limitations**	The trainee must be able to perform and interpret examination in the following situations:<br>Resident level:<br>✓ Chronic hepatopathy follow-up<br>✓ Lesion characterization (HCC, cyst, FNH, haemangioma)<br>✓ Diffuse homogenous hepatic steatosis<br>Fellow level:<br>✓ Lesion characterisation (adenoma and other less common focal lesions)
**Potential risks in case of failure**	Wrong diagnosis<br>Delayed diagnosis<br>Unsuitable biopsy
**Relevant Can-Med roles**	☒Medical expert ☒Leader<br>☒Communicator ☐Health Advocate<br>☒Collaborator ☒Professional
**Knowledge, skills, attitudes and experience**	Knowledge:<br>Resident level:<br>✓ Anatomy of the hepatic segments<br>✓ Complications of chronic hepatopathy<br>✓ Hepatic protocols in CT and MRI<br>✓ Typical features of a simple cyst, haemangioma, FNH<br>✓ Typical imaging features of cirrhosis<br>✓ EASL algorithm for HCC diagnosis<br>✓ LiRADS system<br>Fellow level:<br>✓ Indications/contraindications for CEUS<br>✓ Therapeutic options for HCC<br>Skills:<br>✓ Choose the appropriate imaging modality<br>✓ Provide an imaging protocol adapted to the patient's situation<br>✓ Localize a lesion in the appropriate hepatic segment<br>✓ Use LiRADS/ EASL<br>✓ Use US elastography<br>✓ Perform hepatic CEUS and adapt the injection protocol to the suspected lesion (Fellow level).<br>Attitudes:<br>✓ Adapt the protocol with radiographers<br>✓ Ask for specialized advice when atypical imaging is encountered<br>✓ Provide a clear report and make a diagnosis when typical imaging features are encountered<br>Experience: *(Number of successfully completed examination)*<br>✓ 20 Chronic hepatopathy follow-up US

	✓ 20 Chronic hepatopathy follow-up CT<br>✓ 30 Chronic hepatopathy follow-up MRI<br>✓ 25 Lesion characterization (HCC, cyst, FNH, haemangioma)<br>✓ 10 Diffuse homogenous hepatic steatosis US<br>✓ 10 Diffuse homogenous hepatic steatosis MRI<br>✓ 20 Lesion characterisation (adenoma and other less common focal lesions)		
**Sources of information to support entrustment decisions**	Case based discussion (CBD)<br>Direct observation of practice and procedures (DOPP)<br>Multi-Source Feedback<br>Clinical Work Sampling (CWS) for In-Training Evaluation<br>Case Presentation (CP)		
**Expected level of supervision**	*Resident level*:	3	Resident is allowed to perform without a supervisor in the room, but quickly available if needed, i.e., with indirect, reactive, supervision
	Fellow level:	4	Fellow is allowed to work unsupervised
**Expiration date**	*Resident level*:	4 years	
	Fellow level:	4 years	

<table>
<tr><th colspan="2">EPA 6’. Perform and interpret an exploration for biliary ducts/gallbladder</th></tr>
<tr><td>Specifications and limitations</td><td>The trainee must be able to perform and interpret an imaging examination in the following situations:
Resident level:
✓ Thickening of the gallbladder wall
✓ Obstruction of the common bile duct
Fellow level:
✓ Cholangitis
✓ Post bile duct surgery
✓ Congenital abnormalities of bile ducts
✓ LPAC syndrome</td></tr>
<tr><td>Potential risks in case of failure</td><td>Wrong diagnosis
Pain/sepsis
Tumoral progression</td></tr>
<tr><td>Relevant Can-Med roles</td><td>☒Medical expert ☐Leader
☒Communicator ☐Health Advocate
☒Collaborator ☒Professional</td></tr>
<tr><td>Knowledge, skills, attitudes and experience</td><td><u>Knowledge</u>:
Resident level:
✓ Main aetiologies and features of gallbladder wall thickening
✓ Main aetiologies and features of common bile duct obstruction
Fellow level:
✓ Technical characteristics of MRI for the exploration of bile ducts (Fellow level)
✓ PSC features (US, CT and MRI) and main differential diagnosis (Fellow level)
✓ Main differentials to cholangitis (Fellow level)
✓ Main congenital abnormalities of bile ducts (Fellow level)
✓ Pancreaticobiliary junction abnormalities (Fellow level)
✓ Rare biliary tumours (Fellow level)
✓ Typical features for LPAC syndrome (Fellow level)
<u>Skills</u>:
✓ Choose the most appropriate imaging examination and adapt it according to the clinical problem
✓ Adapt the protocol during examination
<u>Attitudes</u>:
✓ Ask for specialised advice if needed in case of obstruction of the common bile duct
<u>Experience</u>: (Number of successfully completed examination)
✓ 15 Thickening of the gallbladder wall
✓ 10 Obstruction of the common bile duct
✓ 15 Cholangitis
✓ 20 Post bile duct surgery
✓ 15 Congenital abnormalities of bile ducts
✓ 5 LPAC syndrome</td></tr>
<tr><td>Sources of information to support entrustment decisions</td><td>Case based discussion (CBD)
Direct observation of practice and procedures (DOPP)
Multi-Source Feedback
Review of complications
Clinical Work Sampling (CWS) for In-Training Evaluation
Case Presentation (CP)</td></tr>
</table>

**Expected level of supervision**	*Resident level*:	3	Resident is allowed to perform without a supervisor in the room, but quickly available if needed, i.e., with indirect, reactive, supervision
	Fellow level:	4	Fellow is allowed to work unsupervised
**Expiration date**	*Resident level*:	5 years	
	Fellow level:	4 years	

EPA 7’. Perform and interpret a pancreatic imaging examination	
**Specifications and limitations**	The trainee must be able to perform and interpret imaging for:<br>*Resident level:*<br>✓ Acute pancreatitis<br>✓ Chronic pancreatitis<br>*Fellow level:*<br>✓ Common pancreatic cystic lesions<br>✓ Rare pancreatic tumours
**Potential risks in case of failure**	Wrong diagnosis<br>Worsening of the patient's condition
**Relevant Can-Med roles**	☒Medical expert ☐Leader<br>☒Communicator ☐Health Advocate<br>☒Collaborator ☒Professional
**Knowledge, skills, attitudes and experience**	<u>Knowledge</u>:<br>Resident level<br>✓ Atlanta classification for acute pancreatitis<br>✓ Main pancreatitis complications<br>✓ Recommended imaging modality/protocol/timing for exploration<br>✓ Diagnostic criteria for chronic pancreatitis<br>Fellow level:<br>✓ Features of rare pancreatic tumours (SPPT, metastases, acinar tumour, pancreatoblastoma, acinar cystic transformation)<br>✓ Features of auto-immune pancreatitis<br><u>Skills</u>:<br>✓ Choose the most appropriate imaging examination<br>✓ Perform an adapted protocol according to the clinical question and the stage of the disease<br>✓ Identify suggestive features for chronic pancreatitis<br><u>Attitudes</u>:<br>✓ Propose IR in the report when appropriate (fellow level)<br><u>Experience</u>: *(Number of successfully completed examination)*<br>✓ 20 Acute pancreatitis<br>✓ 15 Chronic pancreatitis<br>✓ 20 Common pancreatic cystic lesions<br>✓ 10 Rare pancreatic tumours
**Sources of information to support entrustment decisions**	Case based discussion (CBD)<br>Direct observation of practice and procedures (DOPP)<br>Multi-Source Feedback<br>Clinical Work Sampling (CWS) for In-Training Evaluation<br>Case Presentation (CP)
**Expected level of supervision**	*Resident level*: 3 — Resident is allowed to perform without a supervisor in the room, but quickly available if needed, i.e., with indirect, reactive, supervision<br>*Fellow level:* 4 — Fellow is allowed to work unsupervised
**Expiration date**	*Resident level*: 4 years<br>*Fellow level:* 4 years

EPA 8’. Perform and interpret peritoneal/mesenteric imaging	
**Specifications and limitations**	The trainee must be able to perform and interpret imaging for the following conditions:<br>*Resident level:*<br>✓ Peritoneal metastasis<br>✓ Mesenteric cellular proliferations of infectious and inflammatory origin<br>✓ Tumors of lymphatic origin<br>*Fellow level:*<br>✓ Primary peritoneal tumors<br>✓ Tumors from fatty and connective tissue origin<br>✓ Cystic lesions of extramesenteric origin
**Potential risks in case of failure**	Wrong diagnosis<br>Unsuitable surgery
**Relevant Can-Med roles**	☒Medical expert ☐Leader<br>☐Communicator ☐Health Advocate<br>☒Collaborator ☒Professional
**Knowledge, skills, attitudes and experience**	<u>Knowledge</u>:<br>*Resident level:*<br>✓ Features of peritoneal diseases<br>✓ Features of omental infarction<br>✓ Features of lymphatic diseases<br>✓ Anatomy of the peritoneum and pathways of peritoneal spread of malignant and inflammatory disease<br>✓ Main differential diagnosis (inflammatory, infectious, extra-pelvic endometriosis)<br>*Fellow level:*<br>✓ Features of rare mesenteric tumours<br>✓ Features of rare peritoneal tumours<br><u>Skills</u>:<br>✓ To choose the most appropriate imaging examination and adapt it according to the clinical problem<br><u>Attitudes</u>:<br>✓ Ask for advice in case of rare peritoneal/mesenteric disease<br>✓ Appreciate own limitations and to identify when it is appropriate to obtain assistance<br>✓ Suggest differentials and next step for diagnosis<br><u>Experience</u>: *(Number of successfully completed examination)*<br>✓ 15 examinations for resident level<br>✓ 10 examinations for fellow level
**Sources of information to support entrustment decisions**	Case based discussion (CBD)<br>Direct observation of practice and procedures (DOPP)<br>Multi-Source Feedback<br>Clinical Work Sampling (CWS) for In-Training Evaluation<br>Case Presentation (CP)
**Expected level of supervision**	*Resident level*: 3 — Resident is allowed to perform without a supervisor in the room, but quickly available if needed, i.e., with indirect, reactive, supervision<br>*Fellow level:* 4 — Fellow is allowed to work unsupervised

**Expiration date**	*Resident level*:	4 years
	Fellow level:	4 years

EPA 9'. Assess cancer staging and resectability	
**Specifications and limitations**	The trainee must be able to:<br>• Resident level: diagnose the cancer and assess for metastatic disease<br>• Fellow level: assess loco-regional staging and resectability according to the current staging system<br><br>The concerned cancers are:<br>✓ Small bowel NET<br>✓ Colonic cancer<br>✓ Oesophageal cancer<br>✓ Gastric cancer<br>✓ Liver cancer<br>✓ Rectal cancer<br>✓ Anal cancer<br>✓ Peritoneal metastasis<br>✓ Gallbladder cancer<br>✓ Hilar/biliary cholangiocarcinoma<br>✓ Pancreas adenocarcinoma/NET
**Potential risks in case of failure**	Failure of surgery/interventional radiology<br>Suboptimal treatment<br>Morbidity/mortality
**Relevant Can-Med roles**	☒Medical expert ☐Leader<br>☒Communicator ☐Health Advocate<br>☒Collaborator ☒Professional
**Knowledge, skills, attitudes and experience**	<u>Knowledge</u>:<br>Resident level:<br>✓ NET pathophysiology<br>✓ TNM systems for colorectal and anal cancers<br>✓ Anatomy of the rectum and anal canal<br>✓ Anatomy of the pancreatic region<br>✓ Be aware of online resources to quickly locate current staging systems.<br>Fellow level:<br>✓ NCCN criteria for pancreatic cancer resectability<br>✓ Klatskin classification<br>✓ Peritoneal cancer index (PCI)<br>✓ LiRADS<br><u>Skills</u>:<br>✓ Propose optimal / adapted imaging modality and protocol (radiological or nuclear medicine)<br>✓ Be able to use template reports when the pathology is suitable<br>✓ Choose the most appropriate modality to characterize metastases<br><u>Attitudes</u>:<br>✓ Propose nuclear medicine examination when indicated

	✓ Recommend MDT review when appropriate<br>✓ Appreciate own limitations and to identify when it is appropriate to obtain assistance<br><u>Experience</u>: *(Number of successfully completed examination)*<br>*Resident/Fellow*<br>✓ 5/5 Small bowel NET<br>✓ 10/10 Colonic cancer<br>✓ 5/5 Oesophageal cancer<br>✓ 5/5 Gastric cancer<br>✓ 10/10 Liver cancer<br>✓ 10/10 Rectal cancer<br>✓ 5/5 Anal cancer<br>✓ 10/10 Peritoneal carcinomatosis<br>✓ 5/5 Gallbladder cancer<br>✓ 10/10 Hilar/biliary cholangiocarcinoma<br>✓ 10/10 Pancreas cancer
**Sources of information to support entrustment decisions**	Case based discussion (CBD)<br>Direct observation of practice and procedures (DOPP)<br>Multi-Source Feedback<br>Clinical Work Sampling (CWS) for In-Training Evaluation<br>Case Presentation (CP)
**Expected level of supervision**	*Resident level*: 3 — Resident is allowed to perform without a supervisor in the room, but quickly available if needed, i.e., with indirect, reactive, supervision<br>*Fellow level:* 4 — Fellow is allowed to work unsupervised
**Expiration date**	*Resident level*: 4 years<br>*Fellow level:* 4 years

EPA 10'. Choose and use the appropriate follow-up criteria in abdominal cancers	
**Specifications and limitations**	The trainee must be able to report an onco-digestive follow-up examination using the appropriate criteria according to the pathology and the treatment. The main concerned criteria are:<br>Resident level:<br>✓ Cheson criteria<br>✓ RECIST<br>Fellow level:<br>✓ mRECIST/iRECIST<br>✓ Choi criteria<br>✓ Chun criteria<br>✓ Peritoneal cancer index (PCI)<br>✓ NCCN criteria
**Potential risks in case of failure**	Poor patient care and treatment
**Relevant Can-Med roles**	☒Medical expert ☐Leader<br>☒Communicator ☐Health Advocate<br>☒Collaborator ☒Professional
**Knowledge, skills, attitudes and experience**	Knowledge:<br>✓ The rules for each response criteria/guidelines<br>✓ The CHIP indications<br>Skills:<br>✓ Application for follow-up<br>✓ Choose the appropriate baseline/nadir<br>Attitudes:<br>✓ Search for baseline/nadir and patient's treatment in medical files when needed<br>✓ Communicate with the oncologist about any changes in treatment if necessary<br>✓ Use templates when appropriate<br>✓ Report oncological studies according to international standards applicable to specific situation<br>✓ Make a clear report and a clear oncological conclusion<br>Experience: *(Number of successfully completed examination)*<br>✓ 10 Cheson criteria<br>✓ 10 RECIST<br>✓ 15 mRECIST/iRECIST<br>✓ 10 Choi criteria<br>✓ 10 Chun criteria<br>✓ 10 Peritoneal cancer index (PCI)<br>✓ 15 NCCN criteria
**Sources of information to support entrustment decisions**	Case based discussion (CBD)<br>Direct observation of practice and procedures (DOPP)<br>Multi-Source Feedback<br>Clinical Work Sampling (CWS) for In-Training Evaluation<br>Case Presentation (CP)
**Expected level of supervision**	*Resident level*: 3 — Resident is allowed to perform without a supervisor in the room, but quickly available if needed, i.e., with indirect, reactive, supervision<br>*Fellow level:* 4 — Fellow is allowed to work unsupervised

**Expiration date**	*Resident level*:	4 years
	Fellow level:	4 years

EPA 11’. Perform and interpret an advanced examination of the liver (Fellow level only)	
**Specifications and limitations**	The fellow must be able to perform and interpret the following examinations:<br>✓ Hepatic graft examination (US/CT)<br>✓ CEUS of a liver lesion<br>✓ Elastography of the liver (US/MRI)<br>✓ Liver quantification in MRI (iron, steatosis)<br>✓ Hepatic volumetry<br>✓ MRI with hepatospecific contrast agent
**Potential risks in case of failure**	Wrong diagnosis<br>Missing complications<br>Delay in treatment<br>Supplementary examinations
**Relevant Can-Med roles**	☒Medical expert ☐Leader<br>☒Communicator ☐Health Advocate<br>☒Collaborator ☒Professional
**Knowledge, skills, attitudes and experience**	<u>Knowledge</u>:<br>✓ Physics basis of elastography and iron/steatosis overload<br>✓ Indications for CEUS/elastography/fat/iron quantification<br>✓ Performance and limits of elastography, iron/fat quantification<br>✓ Surgical techniques of hepatic graft<br>✓ Normal and abnormal US values in hepatic graft<br>✓ Pathophysiology of hepatospecific contrast agent<br><br><u>Skills</u>:<br>✓ Adapt CEUS injection to the suspected hepatic lesion<br>✓ Set up and improve MRI sequences to make a diagnosis<br>✓ Use appropriate software (quantification, volumetry)<br>✓ Choose the most suitable contrast material and its optimal use according to the clinical problem<br>✓ Supervise and teach technical staff to ensure that appropriate images are obtained<br><br><u>Attitudes</u>:<br>✓ Look for the patient’s history when necessary (graft surgery)<br>✓ Adapt examination to optimise diagnostic information if there are technical limitations<br>✓ Search in the literature for new studies and guidelines and understand how to appraise their validity and strength<br>✓ Communicate comprehensive results in the report<br>✓ Confidently choose optimal imaging parameters<br>✓ Appreciate own limitations and to identify when it is appropriate to obtain assistance<br><br><u>Experience</u>: *(Number of successfully completed examination)*<br>✓ 20 Hepatic graft examination US<br>✓ 20 Hepatic graft examination CT<br>✓ 20 CEUS of a liver lesion<br>✓ 10 Elastography of the liver US<br>✓ 10 Elastography of the liver MRI<br>✓ 10 Liver quantification in MRI (iron, steatosis)<br>✓ 10 Hepatic volumetry<br>✓ 20 MRI with hepatospecific contrast agent

**Sources of information to support entrustment decisions**	Case based discussion (CBD)<br>Direct observation of practice and procedures (DOPP)<br>Multi-Source Feedback<br>Clinical Work Sampling (CWS) for In-Training Evaluation<br>Case Presentation (CP)		
**Expected level of supervision**	*Fellow level:*	4	Fellow is allowed to work unsupervised
**Expiration date**	*Fellow level:*	4 years	

EPA 12’. Perform and interpret inflammatory bowel disease (IBD) imaging (Fellow level only)	
**Specifications and limitations**	The trainee must be able to perform and interpret a small bowel examination in a patient with known or suspected IBD. These examinations include (Fellow level only):<br>✓ Small bowel CT enterography<br>✓ Small bowel MRI enterography<br>✓ Small bowel US<br>✓ MRI for anal fistula
**Potential risks in case of failure**	Wrong diagnosis<br>Pain for the patient
**Relevant Can-Med roles**	☒Medical expert ☐Leader<br>☒Communicator ☐Health Advocate<br>☒Collaborator ☒Professional
**Knowledge, skills, attitudes and experience**	Knowledge:<br>✓ Differential diagnosis of diffuse or focal small bowel wall thickening according to the clinical context<br>✓ Activity criteria of IBD in MRI and treatment response<br>✓ CT/MRI enterography protocol, including indications and contraindications for antispasmodics.<br>Skills:<br>✓ Perform a small bowel CT enterography<br>✓ Perform a small bowel MRI enterography<br>✓ Check for the quality of the performed examination<br>✓ Perform a small bowel US<br>✓ Choose the most appropriate imaging examination and adapt it according to the clinical problem<br>✓ Supervise and teach technical staff to ensure that appropriate images are obtained<br>Attitudes:<br>✓ Explain the procedure to the patient<br>✓ Communicate with the patient during and after the procedure<br>✓ Integrate the quality of the distension to the conclusion of the report<br><br>Experience: *(Number of successfully completed examination)*<br>✓ 15 Small bowel CT enterography<br>✓ 20 Small bowel MRI enterography<br>✓ 20 Small bowel US<br>✓ 10 MRI for anal fistula
**Sources of information to support entrustment decisions**	Case based discussion (CBD)<br>Direct observation of practice and procedures (DOPP)<br>Multi-Source Feedback<br>Review of complications
**Expected level of supervision**	*Fellow level:* 4 Fellow is allowed to work unsupervised
**Expiration date**	*Fellow level:* 4 years

EPA 13’. React and adapt abdominal imaging in case of pregnancy (Fellow level only)	
**Specifications and limitations**	The fellow must be able to propose the appropriate examination for the diagnosis in case of pregnancy.<br>The fellow must adapt the protocol to the ALARA principle.<br>The fellow must communicate with the patient and the clinician.<br>The fellow must ask the Medical Physics / Biophysics department for advice (if available) when necessary<br>The fellow must ask for a multidisciplinary decision when necessary.
**Potential risks in case of failure**	Stochastic effects on the foetus<br>Deterministic effects on the foetus<br>Wrong diagnosis<br>Parental anxiety
**Relevant Can-Med roles**	☒Medical expert ☐Leader<br>☒Communicator ☐Health Advocate<br>☒Collaborator ☒Professional
**Knowledge, skills, attitudes and experience**	<u>Knowledge</u>:<br>✓ Physics of radiation<br>✓ Radiation dose to the uterus according to the imaging modality and area of the body scanned<br>✓ MRI risks according to the magnetic field<br>✓ Risks and contraindications to contrast injection<br>✓ Risk/benefit ratio of each examination according to the clinical suspicion and context<br><u>Skills</u>:<br>✓ Choose and propose the most appropriate examination and most suitable contrast material for the diagnosis, according to the guidelines and the clinical problem<br>✓ Adapt the protocol to the ALARA principle<br>✓ Confidently choose optimal imaging parameters<br>✓ Supervise and teach technical staff to ensure that appropriate images are obtained<br><u>Attitudes</u>:<br>✓ Communicate with the patient and explain the benefit/risk of the examination<br>✓ Obtain the informed consent of the patient<br>✓ Communicate with the medical team to find the best way to take care of the patient<br>✓ Communicate with the radiographers<br>✓ Provide information to the Medical Physics / biophysics department<br>✓ Ask for specialised gynaecological advice when necessary<br><u>Experience</u>:<br>✓ 5 successfully completed examination
**Sources of information to support entrustment decisions**	Case based discussion (CBD)<br>Direct observation of practice and procedures (DOPP)<br>Multi-Source Feedback<br>Review of complications<br>Case Presentation (CP)
**Expected level of supervision**	*Fellow level:* 4 Fellow is allowed to work unsupervised

**Expiration date**	*Fellow level:*	5 years
